# amoA-encoding archaea and thaumarchaeol in the lakes on the northeastern Qinghai-Tibetan Plateau, China

**DOI:** 10.3389/fmicb.2013.00329

**Published:** 2013-11-12

**Authors:** Jian Yang, Hongchen Jiang, Hailiang Dong, Huanye Wang, Geng Wu, Weiguo Hou, Weiguo Liu, Chuanlun Zhang, Yongjuan Sun, Zhongping Lai

**Affiliations:** ^1^State Key Laboratory of Biogeology and Environmental Geology, China University of GeosciencesWuhan, China; ^2^Key Lab of Salt Lake Resources and Chemistry, Qinghai Institute of Salt Lakes, Chinese Academy of SciencesXining, China; ^3^Department of Geology and Environmental Earth Science, Miami UniversityOxford, OH, USA; ^4^State Key Laboratory of Loess and Quaternary Geology, Institute of Earth Environment, Chinese Academy of SciencesXi’an, China; ^5^State Key Laboratory of Biogeology and Environmental Geology, China University of GeosciencesBeijing, China; ^6^Department of Marine Sciences, University of GeorgiaAthens, GA, USA; ^7^State Key Laboratory of Marine Geology, Tongji UniversityShanghai, China

**Keywords:** *amoA* gene, AEA, Thaumarchaeol, salinity, Qinghai–Tibetan lakes

## Abstract

All known ammonia-oxidizing archaea (AOA) belong to the phylum Thaumarchaeota within the domain Archaea. AOA possess the diagnostic *amoA* gene (encoding the alpha subunit of ammonia monooxygenase) and produce lipid biomarker thaumarchaeol. Although the abundance and diversity of *amoA* gene-encoding archaea (AEA) in freshwater lakes have been well-studied, little is known about AEA ecology in saline/hypersaline lakes. In this study, the distribution of the archaeal *amoA* gene and thaumarchaeol were investigated in nine Qinghai–Tibetan lakes with a salinity range from freshwater to salt-saturation (salinity: 325 g L^-^^1^). The results showed that the archaeal *amoA* gene was present in hypersaline lakes with salinity up to 160 g L^-^^1^. The archaeal *amoA* gene diversity in Tibetan lakes was different from those in other lakes worldwide, suggesting Tibetan lakes (high elevation, strong ultraviolet, and dry climate) may host a unique AEA population of different evolutionary origin from those in other lakes. Thaumarchaeol was present in all of the studied hypersaline lakes, even in those where no AEA *amoA* gene was observed. Future research is needed to determine the ecological function of AEA and possible sources of thaumarchaeol in the Qinghai–Tibetan hypersaline lakes.

## INTRODUCTION

Microbial oxidation of ammonia to nitrite, the first step in nitrification, plays an important role in the global nitrogen cycle. This biogeochemical process is mainly carried out by two groups of microorganisms: ammonia-oxidizing bacteria (AOB) and ammonia-oxidizing archaea (AOA; Nicol and Schleper, 2006), which share a highly divergent homolog of ammonia monooxygenase. The *amoA* gene, encoding the alpha subunit of ammonia monooxygenase, has been widely exploited as a molecular biomarker to study AOB and AOA distributions in various environments (Stahl and de la Torre, 2012). However, it is unknown whether all archaea possessing the *amoA* gene are capable of ammonia oxidation. For example, the uncultivated marine sponge symbiont “*Candidatus* Cenarchaeum symbiosum” possess the *amoA* gene, but there is no evidence for ammonia oxidation (Preston et al., 1996). Accordingly, Dang et al. (2009) proposed a general name “*amoA*-encoding archaea (AEA)” for all *amoA* gene-carrying archaea, which are widely distributed in various ecosystems (Hatzenpichler, 2012; Stahl and de la Torre, 2012 and refs therein).

*amoA*-encoding archaea distributions are controlled by multiple environmental factors (Erguder et al., 2009), among which salinity has been shown to shape AEA diversity in estuaries and saline lakes (Mosier and Francis, 2008; Sahan and Muyzer, 2008; Hu et al., 2010). To date, the highest salinity at which archaeal *amoA* genes have been detected is 36.6 practical salinity units in the water column of the Sargasso Sea (Venter et al., 2004). Thus, it is still unclear whether AEA are present in higher salinity environments and how they respond to changes in salinity from freshwater to hypersaline (up to salt saturation) environments.

In addition to the *amoA* gene, lipids are another type of functional biomarkers for studying AEA distribution in nature. All archaea synthesize a kind of membrane lipids known as isoprenoid glycerol dialkyl glycerol tetraethers (*i*GDGTs; Schouten et al., 2013), which were composed of an isoprenoid carbon skeleton ether bonded to a glycerol-moiety containing 0–4 cyclopentane rings (corresponding to GDGT-0, GDGT-1, GDGT-2, GDGT-3, and GDGT-4, respectively; Pearson and Ingalls, 2013). Additionally, the GDGT “crenarchaeol,” containing four cyclopentyl rings and one cyclohexyl ring was also detected in many marine and lacustrine environments (Castañeda and Schouten, 2011). Up to now, crenarchaeol was only found in the cell membranes of AOA isolates (de la Torre et al., 2008; Schouten et al., 2008; Pitcher et al., 2010), thus many studies proposed crenarchaeol as a characteristic biomarker of AOA (Pearson et al., 2004; Zhang et al., 2006; Pester et al., 2011; Pitcher et al., 2011a; Sinninghe Damsté et al., 2012). Originally, the AOA were thought to belong to the phylum *Crenarchaeota* on the basis of their 16S rRNA genes (Könneke et al., 2005). Subsequently, systematic comparison of 53 ribosomal proteins shared by Archaea, Eukarya, and the genomes of AOA isolates (i.e., *Nitrosopumilus maritimus* and *Nitrososphaera gargensis*) suggested that AOA belong to a separate phylum of the Archaea and should be classified as a new phylum proposed as *Thaumarchaeota* (Brochier-Armanet et al., 2008; Spang et al., 2010). In order to be consistent with the phylum *Thaumarchaeota*, crenarchaeol was renamed thaumarchaeol (Sinninghe Damsté et al., 2012). To date, thaumarchaeol has been detected in different environments, such as marine ecosystems (Sinninghe Damsté et al., 2002; Pitcher et al., 2011a), soils (Weijers et al., 2006), hot springs (Pearson et al., 2004, 2008; Zhang et al., 2006; Pitcher et al., 2009), and lakes (Castañeda and Schouten, 2011). To our knowledge, however, few studies have reported the presence and distribution of thaumarchaeol in hypersaline lakes where salinity is higher than that of seawater.

The Qinghai–Tibetan Plateau is the largest (2 × 10^6^ km^2^) and highest (average ~ 4500 m a.s.l.) plateau on the Earth. It contains thousands of saline/hypersaline lakes, which possess a broad range of environmental gradients such as salinity (from 0.1 to 426.3 g L^-^^1^) and pH (5.4–10.2; Yang et al., 2004; Wu et al., 2006; Dong et al., 2010; Liu et al., 2012; Xiong et al., 2012). So the Qinghai–Tibetan lakes are ideal for assessing AEA diversity and community structure in response to environmental conditions (e.g., salinity). The objectives of this study were: (1) to investigate the abundance and diversity of AEA in Qinghai–Tibetan lakes with different salinities by using an integrated approach including lipids and *amoA* gene-based molecular analysis; (2) to assess how the AEA population correlates with environmental variables such as salinity and pH; and (3) to determine whether thaumarchaeol can be found above the seawater salinity and how AEA respond to salinity change from freshwater to hypersaline.

## MATERIALS AND METHODS

### DESCRIPTION OF STUDY LAKES

**FIGURE 1:**
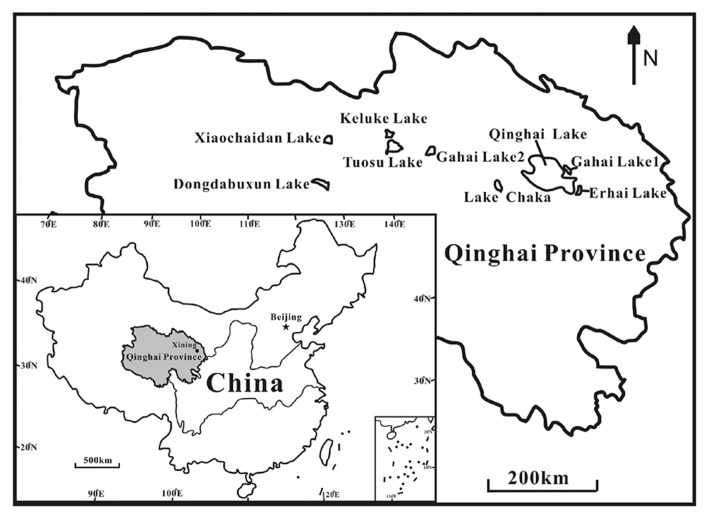
**A geographic map showing the locations of the studied lakes on the Qinghai–Tibet Plateau, China**.

Nine lakes (Keluke Lake, Erhai Lake, Qinghai Lake, Tuosu Lake, Gahai Lake 1, Gahai Lake 2, Xiaochaidan Lake, Dongdabuxun Lake and Lake Chaka) on the Qinghai–Tibetan Plateau were selected for this study (**Figure 1**; **Table 2**). Keluke Lake is situated in the region of Delingha city. It has a surface area of 56.7 km^2^ with the maximum water depth of 13.3 m (Wang and Dou, 1998). Qinghai Lake is the largest saline lake in China, which is located in a structural intermontane depression at the northeastern corner of the Qinghai–Tibetan Plateau. It has an area of 4300 km^2^ and an average water depth of 19.2 m (Dong et al., 2006). Erhai Lake and Gahai Lake 1 are two daughter lakes of Qinghai Lake. Erhai Lake is a freshwater lake with a surface area of ~5 km^2^(Jiang et al., 2010). Gahai Lake 1 is a saline lake with a surface area of ~47.2 km^2^(Jiang et al., 2010). Tuosu Lake is located on the northeastern corner of the Qaidam Basin. It has an area of 165.9 km^2^ and the local average annual temperature is 2–4°C (Wang and Dou, 1998). Gahai Lake 2 is located on the northeastern edge of the Qaidam Basin. It has a surface area of 32 km^2^ with the maximum water depth of 13 m. The lake is situated in an arid climate system (100 mm of rainfall per year; Wang and Dou, 1998). Xiaochaidan Lake is a hypersaline lake located on the northern edge of the Qaidam Basin. It has a surface area of 71.5 km^2^ with the maximum water depth of 0.69 m (Jiang et al., 2009a). Dongdabuxun Lake is a hypersaline lake located in an extremely arid climate region (average rainfall: 24.7 mm per year; Wang and Dou, 1998). It has a surface area of 184.0**–**1001.0 km^2^ with water depth of 0.36–1.02 m. Lake Chaka is a shallow lake with a high salinity of 32.5%. It has a surface area of ~104 km^2^ with average water depth of 2–3 cm (Jiang et al., 2006).

### FIELD MEASUREMENTS AND SAMPLE COLLECTION

Field work was conducted in August 2011. The pH values of the nine lakes were measured with a portable pH meter (PT-10, Sartorius, Germany). Water chemistry (e.g., sulfide, ammonium, and nitrate) was measured with a Hach colorimeter (model CEL 850, Hach Chemical Co., IA, USA). After field measurements, lake surface water samples (250–500 mL) were filtered through 0.2 μm isopore filters (Whatman, UK). The filtrate (~40 mL) was collected into 50-mL falcon tubes for aqueous geochemical analysis. For collection of suspended particulate matter (SPM), lake surface waters (4–20 L) were filtered through 0.7 μm pre-combusted Whatman GF/F glass fiber filters. The biomass-containing filters (both 0.2 and 0.7 μm) were subsequently stored on dry ice. Lake sediments at the water–sediment interface were collected into centrifuge tubes (1.5-mL Eppendorf and 50-mL Falcon tubes for DNA and GDGT samples, respectively) and were immediately frozen on dry ice. For the RNA samples, the sediments were mixed with RNAlater (Ambion, USA) with a water: RNAlater ratio of 1:1 (v:v). All water and sediment samples for DNA/RNA extraction and GDGT analysis were transported to the laboratory on dry ice and stored at -80 and -20°C, respectively until further analyses.

For clarity, these lake names are hereafter abbreviated as follows: KLKL-1-W, EHL-1-W, QHL-14-W, TSL-31-W, GHL1-32-W, GHL2-84-W, XCDL-160W, DDBXL-308-W, and LCK-325-W for Keluke Lake, Erhai Lake, Qinghai Lake, Tuosu Lake, Gahai Lake 1, Gahai Lake 2, Xiaochaidan Lake, Dongdabuxun Lake, and Lake Chaka, respectively. The numbers between “lake name” and “W” indicates salinity (g L^-^^1^) of the lake. KLKL-1-S, EHL-1-S, QHL-14-S, TSL-31-S, GHL1-32-S, GHL2-84-S, XCDL-160-S, DDBXL-308-S, and LCK-325-S are used for the sediments of these lakes.

### MEASUREMENTS OF WATER SALINITY

The concentrations of eight major ions: potassium, sodium, calcium, magnesium, chloride, sulfate, carbonate, and bicarbonate were analyzed in the laboratory according to the Manual of Analytical and Testing Department in the Institute of Salt Lakes, Chinese Academy of Science (Analytical Lab of the Qinghai Institute of Salt Lakes, Chinese Academy of Sciences, 1988). Salinity was calculated by summing the concentrations of these eight ions.

### THAUMARCHAEOL ANALYSIS

GF/F filters and sediments (~5 g per sample) were freeze-dried and extracted according to the procedures described previously (Wang et al., 2013). Briefly, samples were ultrasonically extracted three times using a single phase solvent mixture including MeOH, dichloromethane (DCM), and phosphate buffer (pH 7.4; 2:1:0.8, v/v/v) following a previous procedure (Bligh and Dyer, 1959). Samples were centrifuged (5 min, 2500 rpm) and the extract was collected into another tube. This procedure was repeated three times. DCM and phosphate buffer were added to the combined extract at 1:1:0.9 (v/v/v) to achieve phase separation, after which the bottom DCM phase (containing lipids) was collected into a 40 mL glass tube. The resulting aqueous phase was rinsed twice with DCM and all DCM fractions were collected into a glass tube. Subsequently, the DCM phase containing the total Bligh–Dyer extract (BDE) was dried under N_2_. To quantify thaumarchaeol a known amount of a C_46_ internal standard (Huguet et al., 2006) was added to the BDE which was then dissolved in DCM. The resulting mixture was divided into two aliquots: one was dried under N_2_, re-dissolved in hexane/isopropanol (99:1 v/v), and filtered through a 0.45 μm polytetrafluoroethylene (PTFE) filter for analysis of archaeal core lipids (CLs); and the other was subject to acid hydrolysis, and the extracted organic phase was re-dissolved in hexane/isopropanol (99:1, v/v) and filtered through a 0.45 μm PTFE filter for archaeal total lipid analysis. The difference in yield of archaeal lipids between the hydrolyzed and non-hydrolyzed fractions is considered to be the archaeal polar lipids (PLs; Zhang et al., 2011). The thaumarchaeol was determined by using high performance liquid chromatography (HPLC)–atmospheric pressure chemical ionization (APCI)–mass spectrometry (MS) at Tongji University following a method slightly modified from previous studies (Hopmans et al., 2004; Schouten et al., 2007). An aliquot (5 μl) of sample was injected and separation was achieved with an Alltech Prevail Cyano Column (150 mm × 2.1 mm, 3 μm). The elution gradient was: isocratic (5 min) at 99% hexane/1% isopropanol followed by a linear gradient to 1.8% propanol in 45 min at a constant flow rate of 0.2 ml min^-^^1^. Quantification was achieved by peak area integration of [M + 1]^+^ ions in the extracted ion chromatogram in comparison with the C_46_ internal standard. The detection limit was 0.8 pg (Zhang et al., 2011).

### NUCLEIC ACIDS EXTRACTION

DNA was extracted from biomass-containing filters and lake sediments by using FastDNA Pro soil-direct kits (MP Biomedicals, OH, USA) according to the manufacturer’s instructions. The *amoA* transcripts have been recovered from Qinghai Lake (Jiang et al., 2009b) and oceans (with salinity higher than Gahai Lake 1; Pitcher et al., 2011b). In order to test whether the *amoA* gene transcripts can be recovered from saline lakes (with salinity higher that of seawater), sediment samples from Gahai Lake 2 and Xiaochaidan Lake were selected for RNA extraction using FastRNA Pro soil-direct kits (MP Biomedicals) according to the manufacturer’s protocol. DNA-based *amoA* gene PCR was not successful in Lake Chaka, so RNA was not extracted from the sediment of Lake Chaka). The extracted raw RNA was digested with the use of RNase-free DNase I (Takara, Japan). The DNase-digested RNA samples were checked for potential geonomic DNA contamination by PCR amplification with the AOA-specific primer set (see “PCR Amplification and Phylogenetic Analysis”). The checked RNA samples were reverse-transcribed into cDNA using the Promega AMV reverse transcription system (Promega Corporation, Madison, WI, USA) as previously described (Yang et al., 2012). Double distilled water served as the template in negative controls for the cDNA synthesis and downstream PCRs (Jiang et al., 2010).

### QUANTITATIVE PCR

Quantitative PCR (qPCR) was used to determine the abundances of the archaeal 16S rRNA and *amoA* genes in the waters and sediments of the nine lakes with the primer sets of Arch349F (5′-GYG CAS CAG KCG MGA AW-3′)/Arch806R (5′-GGA CTA CVS GGG TAT CTA AT-3′; Takai and Horikoshi, 2000), and Arch-amoAF (5′-STAATGGTCTGGCTTAGACG-3′)/Arch-amoAR (5′-GCGGCCATCCATCTGTATGT-3′; Francis et al., 2005), respectively. qPCRs were performed in a reaction volume of 20 μL, containing 10 μL of 2 × SYBR^®^ Premix Ex Taq^TM^ (Takara), 0.4 μM of each primer, 0.4 μL of ROX reference dye II (50×), and 1 μL of soil DNA. qPCRs were performed on an ABI7500 real-time PCR system (Applied Biosystems, Carlsbad, CA, USA). The qPCRs were performed with the following conditions: 95°C for 30 s, followed by 40 cycles (5 s at 95°C for denaturing, 34 s for annealing at 53°C for the archaeal 16S rRNA and *amoA* genes, and 60 s at 72°C). A dissociation stage was added to yield a dissociation curve after the cycling amplification step. Standard curves were obtained by using serial dilutions (10^1^ to 10^7^ copies) of plasmids (pGEM-T) containing cloned archaeal 16S rRNA and *amoA* genes. The data were used to create standard curves correlating the *C*_t_ values with the archaeal 16S rRNA and *amoA* gene copy numbers. Linear plots (not shown) between the *C*_t_ value and log (copy numbers/reaction) were obtained with correlation coefficients of *R*^2^ > 0.99. PCR efficiencies were 90–95%. The quality and length of the qPCR products were checked by dissociation curve analysis and 1% agarose gel electrophoresis. The qPCR results were expressed as gene copies per gram (copies g^-^^1^) for sediments and gene copies per milliliter (copies mL^-^^1^) for water samples.

### PCR AMPLIFICATION AND PHYLOGENETIC ANALYSIS

Five lakes (Erhai Lake, Gahai Lake 1, Gahai Lake 2, Xiaochaidan Lake, and Lake Chaka) were selected for the AEA *amoA* gene diversity analysis. Two primer sets of Arch-amoAF/Arch-amoAR (Francis et al., 2005) and CrenamoA23f (5′-ATGGTCTGGCTWAGACG-3′)/CrenamoA616r (5′-GCCATCCATCTGTATGTCCA-3′; Tourna et al., 2008) were used to PCR-amplify the archaeal *amoA* genes from the extracted DNA and synthesized cDNA. Each PCR mixture (25 μL reaction volume) contained the following ingredients: 2.5 μL 10× buffer (Takara), 2 μL deoxynucleoside triphosphate (dNTP; a 2.5 mM dNTP; Takara), 16.2 μL sterilized ultra pure water (Millipore), 1 μL bovine serum albumin (BSA; Takara), 1 μL each primer (10 pmole), and 0.3 μL rTaq DNA polymerase (Takara). PCR conditions were same as those described previously (Francis et al., 2005; Tourna et al., 2008). The resulting PCR products (635 and 629 bp from the primer sets of Arch-amoAF/Arch-amoAR and CrenamoA23f/CrenamoA616r, respectively) were examined on 1% agarose gel and no bands were observed for PCR negative controls (with distilled water as the template). PCRs failed for the DNA samples from Lake Chaka (with 325 g L^-^^1^ salinity). Transcription of archaeal *amoA* gene was shown to occur in Erhai Lake and Gahai Lake 1 in our previous study (Jiang et al., 2009b). Therefore cDNA synthesis was performed only on the samples from Gahai Lake 2 and Xiaochaidan Lake. However, Xiaochaidan Lake cDNA sample was only successfully amplified with primer Arch-amoAF/Arch-amoAR. The appropriate bands were excised and PCR gels were purified with Agarose Gel DNA purification Kit (Takara). The purified PCR products were ligated into the pGEM-T vector (Promega Inc.) and transformed into *Escherichia coli* JM109 competent cells (Takara) according to the manufacturer’s instructions. The transformed cells were spread on Luria–Bertani plates containing 100 μg mL^-^^1^ of ampicillinsodium, 80 μg mL^-^^1^ of X-Gal (5-bromo-4-chloro-3-indolyl-β-D-galactopyrano-side), and 0.5 mM IPTG (isopropyl-β-D-thiogalactopyranoside) and cultivated overnight at 37°C.

Sixteen (eight for each primer set) DNA clone libraries were constructed from the following samples: EHL-1-W, GHL1-32-W, GHL2-84-W, XCDL-160-W, EHL-1-S, GHL1-32-S, GHL2-84-S, and XCDL-160-S. One cDNA clone library (XCDL-160-SR) was constructed for the sediment from Xiaochaidan Lake. Around 30–40 randomly selected clones per sample were analyzed for the insert *amoA* gene sequences. Positive clones were sequenced using M13F with the BigDye Terminator version 3.1 chemistry (Applied Biosystems, Foster City, CA, USA) on an ABI 3730 automated sequencer.

The obtained raw nucleotide sequences were checked and trimmed manually by using the BioEdit program^2^. The sequences of poor quality were removed from further analysis. The operational taxonomic units (OTUs) of the *amoA* gene clone sequences were determined based on a cutoff value of 98% by using nearest neighbor algorithm in the DOTUR program (Schloss and Handelsman, 2005). The saturation of the sampled clones from each *amoA* gene clone library was assessed by calculating the coverage (*C*) values as follows: *C* = 1 - (*n*1/*N*), where *n*1 is the number of OTUs that occurred only once in the clone library and *N* is the total number of analyzed clones (Jiang et al., 2006). One representative sequence was selected from each OTU for phylogenetic analysis. Closest references of the *amoA* gene were retrieved from the GenBank^3^ using BLAST. Maximum likelihood trees were constructed from the *amoA* gene sequences obtained in this study and their references (Pester et al., 2012) with the use of the MEGA program version 5.0 (Tamura et al., 2011), and were assessed using 1000 bootstrap replications. The nucleotide sequences obtained in this study were deposited in the GenBank database under accession numbers JX488399–JX488453 and KF606897–KF606927.

### STATISTICAL ANALYSIS

Diversity indices were calculated by using DOTUR. LIBSHUFF analysis was performed to discern any similarity of the *amoA* gene composition among the samples according to the methods described previously (Jiang et al., 2006). Mantel test was performed to assess the correlations between *amoA* gene populations and environmental factors according to the procedures described previously (Yang et al., 2013). Briefly, the biotic matrices of *amoA* gene composition were constructed with the Bray–Curtis distance defined as follows: Bray–Curtis distance = 1 - *d*, where *d* refers to the Bray–Curtis similarity index, and the abiotic matrices of environmental variables were constructed using the Euclidean distance.

Cluster analysis was performed to compare differences in AEA communities between the studied Tibetan lakes and other lakes and saline environments worldwide (Francis et al., 2005; Dang et al., 2009; Herrmann et al., 2009; Jiang et al., 2009b; Kalanetra et al., 2009; Pouliot et al., 2009; Llirós et al., 2010; Wu et al., 2010; Auguet et al., 2011; **Table 1**). In order to avoid any bias resulting from different primers, those archaeal *amoA* gene sequences derived from the same primer set, Arch-amoAF/Arch-amoAR (Francis et al., 2005) were included in the cluster analysis. The combined *amoA* gene sequences (~635 bp) were subjected to OTU identification using the DOTUR program (Schloss and Handelsman, 2005). The Jaccard similarity matrices were made and topology trees were constructed using the PAST software package^4^.

**Table 1:** Summary of the AEA *amoA* gene sequences used for the comparison between this and other studies (the AEA *amoA* gene sequences were derived from the primer set of Francis et al. (2005), and the length of the *amoA* gene sequences was 635 bp.

Location	Lake name (sample type)	No. of clones or sequences	Reference
Denmark	Lake Hampen (sediment)	306	Herrmann et al. (2009)
	Lake Kalgård (sediment)		
	Lake Grane Langsø (sediment)		
	Lake Søby (sediment)		
	Lake Almind (sediment)		
Spain	Lake Llebreta (water)	Total 40 DGGE sequences	Auguet et al. (2011)
	Lake Llong (water)	
	Lake Redo’ AT (water)		
Congo	Lake Kivu (water)	14(DGGE bands)	Llirós et al. (2010)
Canada	High Arctic lake A (water)	22	Pouliot et al. (2009)
	High Arctic lake C1 (water)	28	
China	Qinghai Lake water (QLW1-0)	22	Jiang et al. (2009b)
	Qinghai Lake sediment (QLS1-30)	16	
	Lake Taihu (sediment)	106	Wu et al. (2010)
Miscellaneous	Antarctic coast (water)	119	Kalanetra et al. (2009)
	Arctic Ocean (water)		
	Monterey Bay (water)	47	Francis et al. (2005)
	The Eastern Tropical North Pacific (water)	26	
	Black Sea (water)	70	
	Elkhorn Slough (sediment)	69	
	San Francisco Bay (sediment)	50	
	Bahïa del Tbóari, Mexico (sediment)	64	
	Huntington Beach, CA (sediment)	24	
	Oak ridge (soil)	27	
	The deep-sea sediments of the tropical West Pacific continental margin (sediment)	242	Dang et al. (2009)

## RESULTS

### WATER CHEMISTRY OF THE STUDIED LAKES

The salinities of the investigated lakes were 0.7, 1.0, 14.2, 31.3, 31.9, 84.0, 160.4, 307.6, 325.0 g L^-^^1^ for Keluke Lake, Erhai Lake, Qinghai Lake, Tuosu Lake, Gahai Lake 1, Gahai Lake 2, Xiaochaidan Lake, Dongdabuxun Lake, and Lake Chaka, respectively (**Table 2**). The pH was 7.0–9.4. The concentrations of sulfide ranged from 0.0 to 0.2 (mg L^-^^1^; **Table 2**). The concentrations of major cations and anions of the investigated lake waters ranged as follows (mg L^-^^1^): K^+^ (6.5–2163.0), Na^+^ (135.2–107460.3), Ca^2^^+^ (17.7–1711.0), Mg^2^^+^ (53.6–24700.0), ${SO}_{4}^{2-}$ (117.9–17099.8), Cl^-^ (206.2–196231.9), ${CO}_{3}^{2-}$ (0.0–515.4), ${HCO}_{3}^{2-}$ (149.8–824.1) ${NH}_{4}^{+}$ (0.4–1.2), and ${NO}_{3}^{-}$ (0.2–1.4; **Table 2**).

**Table 2:** Geographic and geochemical parameters of the nine lakes on the Qinghai–Tibetan Plateau, China (BDL indicates below the detection limit, 0.01 mg L^-1^).

	Keluke lake	Erhai lake	Qinghai lake	Tuosu lake	Gahai lake 1	Gahai lake 2	Xiaochaidan lake	Dongdabuxun lake	Lake Chaka
GPS location (N/E)	37°18.7′/	36°34.1′/	36°38.0′/	37°11.6′/	36°58.1′/	37°7.8′/	37°28.8′/	37°28.8′/	36°45.0′/
	96°54.1′	100°44.3′	100°6.9′	96°53.3′	100°35.9′	97°46.9′	95°26.2′	95°26.2′	99°4.8′
Salinity (g L^-^^1^)	0.7	1.0	14.2	31.3	31.9	84.0	160.4	307.6	325.0
pH	8.8	9.4	9.1	8.8	8.9	8.4	8.4	7.0	7.8
Sulfide (mg L^-^^1^)	BDL	0.1	BDL	BDL	0.1	BDL	0.1	0.2	0.0
**Concentration of major ions (mg L^-^^1^**)
K^+^	6.5	9.0	269.2	326.9	613.0	452.9	920.0	2163.0	2089.3
Na^+^	135.2	218.8	3993.0	8087.0	9384.0	26770.0	54691.0	77950.0	107460.3
Ca^2^^+^	38.5	20.0	17.7	32.4	22.8	392.0	625.0	1711.0	823.2
Mg^2^^+^	53.6	68.5	824.1	2107.0	1467.0	4097.0	2588.7	24700.0	9740.8
${SO}_{4}^{2-}$	140.4	117.9	2188.0	6454.0	6058.0	10790.0	28735.2	4462.0	17099.8
Cl^-^	206.2	231.1	5625.7	13129.5	13034.7	41059.4	72063.4	196231.9	187633.2
${CO}_{3}^{2-}$	0.0	46.6	405.0	416.0	515.4	112.9	366.1	BDL	BDL
${HCO}_{3}^{-}$	149.8	268.5	854.1	767.9	824.1	312.2	380.1	359.6	153.1
${NO}_{3}^{-}$	0.5	0.2	0.4	0.4	0.5	0.6	0.5	1.1	1.4
${NH}_{4}^{+}$	1.0	1.2	1.0	0.8	0.6	0.5	0.6	0.4	0.4

### ABUNDANCE OF THAUMARCHAEOL

**FIGURE 2:**
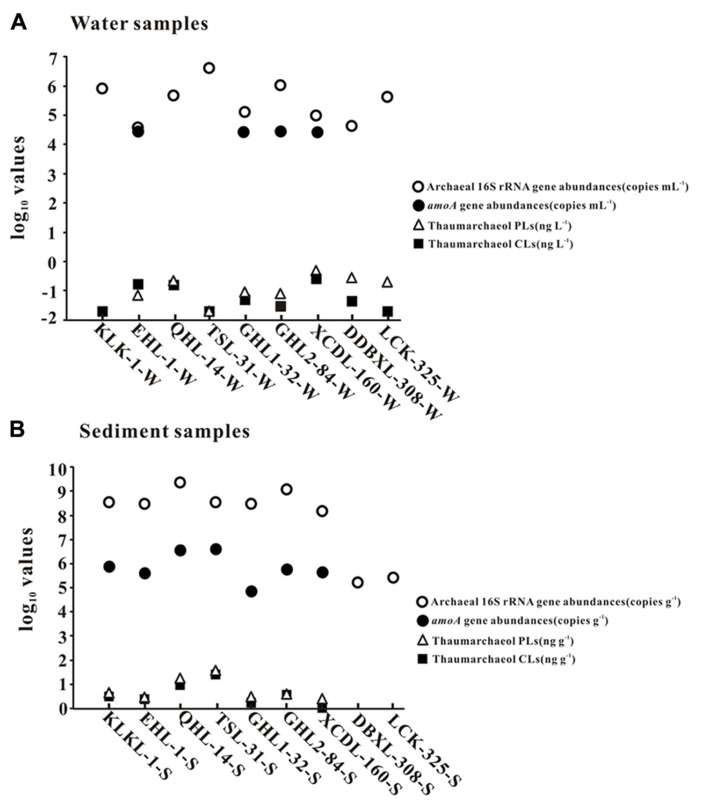
**The abundances (copies per gram of sediment or copies per milliliter of water) of total archaeal 16S rRNA and *amoA *genes and thaumarchaeol concentrations (nanogram per liter of water and nanogram per gram of sediment).** Panels **(A)** and **(B)** are for waters and sediments, respectively.

In the lake waters, thaumarchaeol concentration ranged from 0.0 to 0.3 ng L^-^^1^ for CLs and from 0.0 to 0.5 ng L^-^^1^ for PLs; in the lake sediments, thaumarchaeol concentrations ranged from 0.1 to 25.2 ng g^-^^1^ for CLs and 0.5 to 37.5 ng g^-^^1^ for PLs (**Figure 2**). The highest thaumarchaeol concentrations were observed in the sediment of Tuosu Lake (salinity: 31 g L^-^^1^): 25.2 and 37.5 ng g^-^^1^ for CLs and PLs, respectively (**Figure 2**).

### ABUNDANCE OF ARCHAEAL 16S rRNA AND *amo*A GENES

**FIGURE 3:**
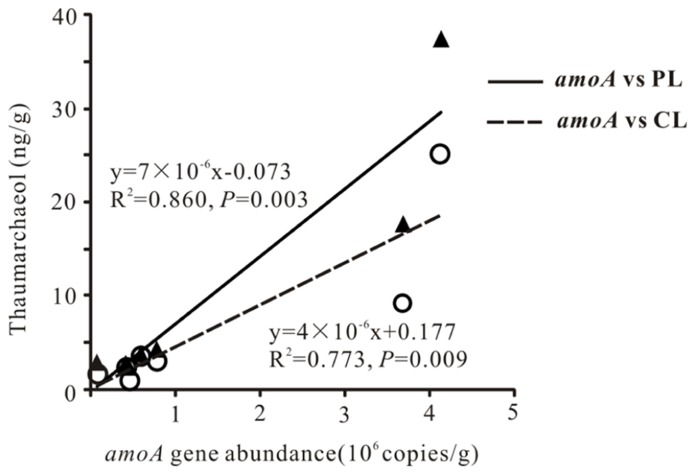
**Scatter plot showing a correlation between sediment *amoA* gene copies and thaumarchaeol**.

Total archaeal 16S rRNA gene abundance ranged from 3.86 × 10^4^ to 4.15 × 10^6^ copies mL^-^^1^ and from 1.66 × 10^5^ to 2.33 × 10^9 ^copies g^-^^1^ for the waters and sediments, respectively (**Figure 2**). The *amoA* gene abundance ranged from 2.81 × 10^4^–3.07 × 10^4^ copies mL^-^^1^ and 7.34 × 10^4^–4.13 × 10^6^copies g^-^^1^ for the waters and sediments, respectively (**Figure 2**). The highest water and sediment 16S rRNA archaeal abundances were observed in the water of Tuosu Lake (4.15 × 10^6^copies mL^-^^1^) and the sediment of Qinghai Lake (2.33 × 10^9^ copies g^-^^1^); whereas the highest water and sediment archaeal *amoA* gene abundances were observed in the water of Gahai Lake 1 (3.07 × 10^4^ copies mL^-^^1^) and the sediment of Tuosu Lake (4.13 × 10^6^ copies g^-^^1^). In addition, the *amoA* gene abundance exhibited a significant correlation (CL: *r* = 0.879, *P* = 0.009; PL: *r* = 0.928, *P* = 0.003) with thaumarchaeol abundance for the sediment samples (except Lake Chaka, where no *amoA* gene abundance data were available; **Figure 3**).

### *amoA* GENE PHYLOGENETIC ANALYSIS

A total of 283 (187 and 96 from the two primer sets of Arch-amoAF/Arch-amoAR and CrenamoA23f/CrenamoA616r, respectively) *amoA* gene clone sequences were obtained (**Table 3**) The *amoA* gene clone sequences derived from the primer set of Arch-amoAF/Arch-amoAR were clustered into 55 OTUs: 12, 3, 7, and 9 for the water samples and 4, 2, 8, and 10 for the sediment samples of Erhai Lake, Gahai Lake 1, Gahai Lake 2, and Xiaochaidan Lake, respectively (**Table 3**). The coverage ranged from 72 to 100% for the *amoA* gene clone libraries (**Table 3**). Diversity indices were 2.0–19.0, 0.1–0.7, and 0.5–2.2 for Chao1, Simpson, and Shannon–Weaver, respectively (**Table 3**).

**Table 3:** Ecological estimates of AEA *amoA* gene clone sequences amplified with primer sets of Arch-amoAF/Arch-amoAR (Francis et al., 2005) and CrenamoA23f/CrenamoA616r (Tourna et al., 2008; as indicated by the numbers before and after the backlashes, respectively).

Clone libraries	EHL-1-W	QHL-14-W^*^	GHL1-32-W	GHL2-84-W	XCDL-160-W	EHL-1-S	QHL-14-S^*^	GHL1-32-S	GHL2-84-S	XCDL-160-S
Library size (no.of clones)	24/12	19/ND	23/9	20/8	24/11	25/13	24/ND	24/12	23/16	24/15
Coverage (%)	72/83	100/ND	100/100	90/100	83/82	9692	100/ND	100/100	87/87	7993
No. of observed OTUs (98% cutoff)	12/5	3/ND	3/3	7/1	9/5	4/4	6/ND	2/1	8/6	10/6
Chao 1	19.0/5.5	NA/ND	3.0/3.0	7.3/NA	12.0/5.5	4.0/4.0	NA/ND	2.0/NA	9.5/6.3	15.0/6.5
Simpson’s diversity index (*D*)	0.1/0.2	NA/ND	0.3/0.3	0.2/NA	0.2/0.2	0.3/0.4	NA/ND	0.7/NA	0.2/0.2	0.1/0.2
Shannon–Weaver’s diversity index (*H*)	2.2/1.4	1.0/ND	1.0/1.1	1.8/NA	1.9/1.5	1.1/1.1	1.7/ND	0.5/NA	1.9/1.5	2.0/1.6

* Clone libraries QHL-W and QHL-S corresponded to QLW1-0 and QLS-30 in Jiang et al. (2009b), respectively; ND, not determined; NA, not available.

In comparison, the *amoA* gene diversity derived from CrenamoA23f/CrenamoA616r was lower than that from the Arch-amoAF/Arch-amoAR primer set. These *amoA* gene clone sequences fell into 31 OTUs: 5, 3, 1, and 5 for the water samples, and 4, 1, 6, and 6 for the sediment samples of Erhai Lake, Gahai Lake 1, Gahai Lake 2, and Xiaochaidan Lake, respectively (**Table 3**). The coverage ranged from 82 to 100% for the *amoA* gene clone libraries (**Table 3**). Diversity indices were 3.0–6.5, 0.2–0.4, and 1.1–1.6 for Chao1, Simpson, and Shannon–Weaver, respectively (**Table 3**).

**FIGURE 4:**
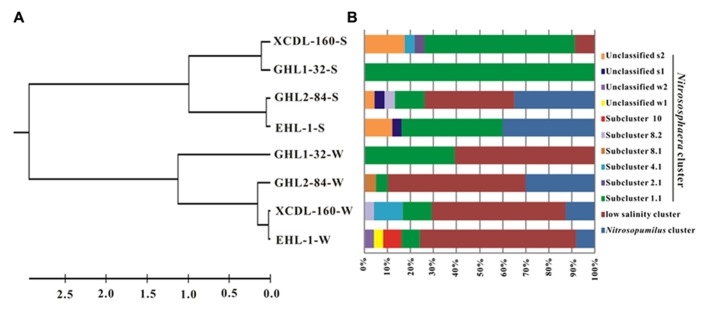
**(A)** Clustering of the different *amoA* gene clone libraries based on ΔCxy values determined from the LIBSHUFF analysis. The tree was constructed with the unweighted-pair group method using average linkages in MEGA 5. The parameter ΔCxy in the LIBSHUFF analysis represents the difference in coverage of any two clone libraries (the larger ΔCxy, the greater dissimilarity between the given clone libraries). The software for the analysis was available at  **(B)** Schematic figure showing the frequencies of OTUs (at 98% nucleotide cutoff) affiliated with major phylogenetic groups in the *amoA* gene clone libraries.

The LIBSHUFF analysis showed that the archaeal *amoA* gene clone libraries were grouped into two separate clusters (one each for the waters and sediments, respectively; *P*-value < 0.01; **Figure 4A**).

**FIGURE 5:**
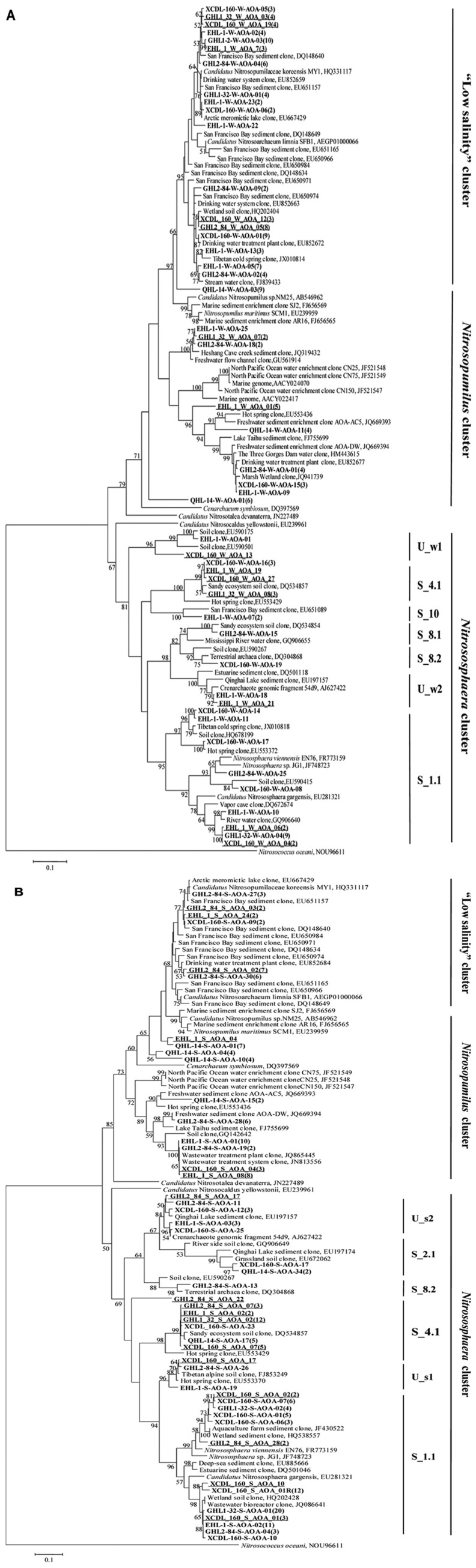
**Maximum likelihood tree (partial sequences, 635 or 629 bp) showing the phylogenetic relationships of the *amoA* gene clone sequences obtained in this study to their closely related sequences from the GenBank database.** One representative clone type within each OTU is shown, and the number of clones within each OTU is shown in parentheses. If there is only one clone sequence within a given OTU, the number “1” is omitted. The sequences from this study are bolded, and they are coded as follows for the example of XCDL-S-AOA-17: *amoA* sequences of clone no. 17 from the Xiaochaidan Lake sediment. Clone libraries QHL-14-W and QHL-14-S were corresponded to QLW1-0 and QLS-30 in Jiang et al. (2009b), respectively. The “R” symbol in some clone names denotes RNA-based (cDNA) clones. The underlined clone sequences were derived from the CrenamoA23f /CrenamoA616r primer set. The classification system of Pester et al. (2012) was employed. The letters “S” and “U” in the cluster names indicated “subcluster” and “unclassified.” The scale bars indicate the Jukes–Cantor distances. Bootstrap values of (1000 replicates) >50% are shown. The bacterial *amoA* gene from *Nitrosococcus oceani* was used as outgroup. Panels **(A)** and **(B)** are for waters and sediments, respectively.

The *amoA *gene clone sequences obtained from the waters were grouped into the *Nitrososphaera* clusters (subcluster 1.1, 4.1, 8.1, 8.2, 10, and unclassified w1 and w2), *Nitrosopumilus* clusters and a “low salinity” cluster (Mosier and Francis, 2008; **Figures 4B and 5A**). The “low salinity” cluster was the predominant component (accounting for 62.6%) in the total water *amoA* gene clone sequences. The *amoA* gene clone sequences in the “low salinity” cluster had high an identity (~98%) with the clones from the San Francisco Bay estuary (Francis et al., 2005; Mosier and Francis, 2008) and a low-salinity ammonia-oxidizing archaeon “*Candidatus* Nitrosoarchaeum limnia” (Blainey et al., 2011). The *amoA* gene clone sequences in the *Nitrosopumilus* cluster were closely related (approximately 98% identity) to the clones retrieved from diverse environments, such as drinking water treatment plant (van der Wielen et al., 2009), Tibetan marsh wetland (unpublished), freshwater flow channel (Herrmann et al., 2011), waters near the Three Gorges Dam of Yangtze River (Huang et al., 2011), and freshwater sediment enrichment clones (AOA-AC5 and AOA-DW; French et al., 2012b). The *amoA* gene clone sequences in the *Nitrososphaera* cluster showed close relatedness (~98% identity) to clones from soils (unpublished), estuary sediments (Beman and Francis, 2006), Qinghai Lake sediments (Jiang et al., 2009b), and a AOA isolate *Nitrososphaera viennensis* EN76 (Tourna et al., 2011).

For the sediment samples, the obtained *amoA* clone sequences were grouped into the *Nitrososphaera* clusters (subcluster 1.1, 2.1, 4.1, 8.2, and unclassified s1 and s2), *Nitrosopumilus* clusters and “low salinity” Cluster (**Figures 4B** and **5B**), with the *Nitrososphaera *1.1 cluster being the dominant (55.2% of the obtained sediment AOA *amoA* gene clone sequences). The *amoA* gene clone sequences in the *Nitrososphaera *1.1 cluster were closely related (approximately 98% identity) to clones retrieved from diverse environments, such as waste water bioreactors (NCBI database), littoral wetland soils (Wang et al., 2012), wetland sediments (Wang et al., 2011), aquaculture farm sediments (Dang et al., 2010), alpine soils (Zhang et al., 2009), sandy soils (Leininger et al., 2006), and Qinghai Lake sediments (Jiang et al., 2009b). The *amoA* gene clone sequences in the *Nitrosopumilus* cluster showed a high similarity (~98%) to clones from Lake Taihu sediment (Wu et al., 2010), drinking water treatment plants (van der Wielen et al., 2009), and freshwater sediment enrichment clones (AOA-AC5 and AOA-DW; French et al., 2012b). The *amoA* gene clone sequences in the “low salinity” cluster had high an identity (~98%) with the clones from the San Francisco Bay sediment (Francis et al., 2005; Mosier and Francis, 2008) and drinking water treatment plants (van der Wielen et al., 2009). In addition, the cDNA-based AEA *amoA* gene clone sequences (*n* = 12) from Xiaochaidan Lake were grouped into one OTU and were affiliated with S_1.1 subcluster within *Nitrososphaera* (**Figure 5B**).

### STATISTICAL ANALYSIS

The abundances of *amoA* gene and thaumarchaeol did not show any significant correlations with any of the measured environmental variables, such as pH, salinity, salinity-related ions, ammonium, nitrate, and sulfide (data not shown). No significant correlation was observed between the *amoA* gene composition (at the 98% similarity OTU level) and water chemistry (data not shown). The simple Mantel test showed no significant correlation between AEA composition and salinity at the 98% similarity OTU level (water samples: *r* = -0.491, *P* = 0.872 or sediment samples: *r *= 0.587, *P* = 0.132).

**FIGURE 6:**
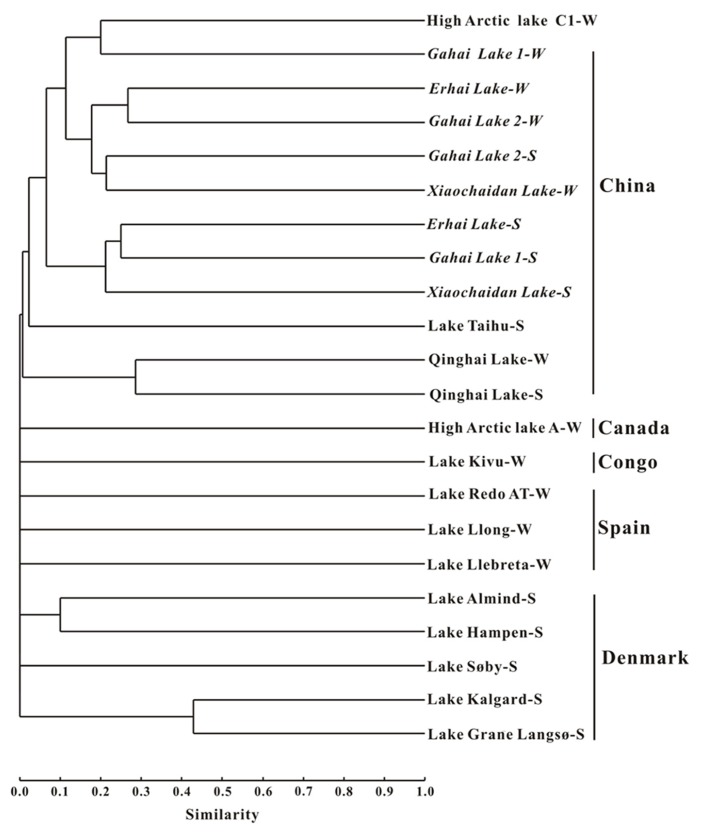
**Jaccard similarity-based cluster analysis of the *amoA* gene communities in different lakes worldwide.** The archaeal *amoA* gene sequences obtained in this study were combined with those previously reported for Qinghai Lake (Jiang et al., 2009b), Lake Taihu (Wu et al., 2010), high arctic lake (Pouliot et al., 2009), Lake Kivu (Llirós et al., 2010), Spanish lakes (Auguet et al., 2011), and Danish lakes (Herrmann et al., 2009). The numbers of clones from each location are given in **Table 1**. “W” and “S” indicate water and sediment samples, respectively.

**FIGURE 7:**
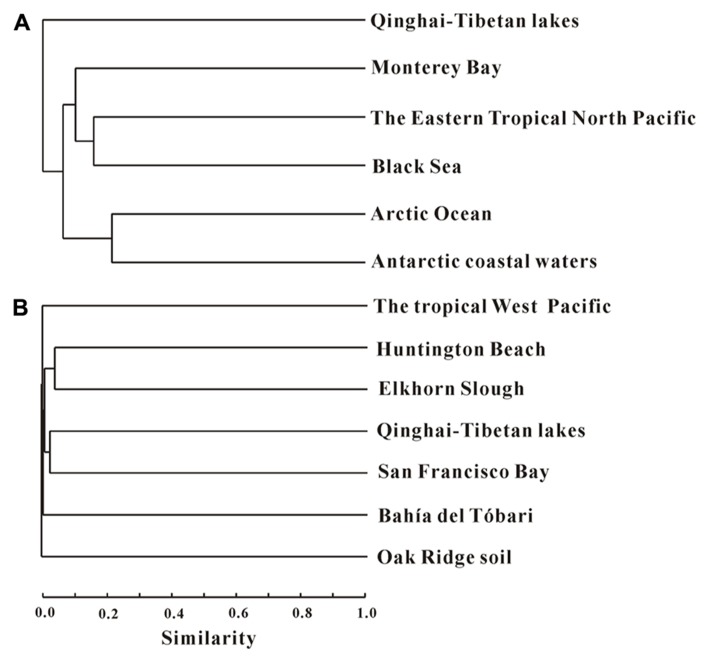
**Jaccard similarity-based cluster analysis of the *amoA* gene communities in different habitats worldwide.** Panels **(A)** and **(B)** are for water and sediment/soil samples, respectively. **(A)** The archaeal *amoA* gene sequences obtained in this study were compared with those previously reported in the waters of Monterey Bay, the Eastern Tropical North Pacific, and the Black Sea (Francis et al., 2005), and Arctic Ocean and Antarctic coasts (Kalanetra et al., 2009). **(B)** The archaeal* amoA* gene sequences obtained in this study were compared with those previously reported in the sediments from Elkhorn Slough, and Huntington Beach of California, Bahïa del Tbóari of Mexico, and the San Francisco Bay (Francis et al., 2005), the tropical West Pacific Continental Margin (Dang et al., 2009), and an Oak Ridge soil (Francis et al., 2005). The numbers of clones from each location are given in **Table 1**.

Cluster analysis showed that the AEA communities in the lakes (including the saline/hypersaline lakes in this study and the freshwater lakes in other studies) within China were grouped into one cluster, separated from other freshwater lakes (except for the high Arctic Lake C1) around the world (e.g., Canada, Congo, Spain, Denmark; **Figure 6**). In addition, the AEA communities in Tibetan lakes exhibited little similarity to those in other saline habitats, such as the waters of Monterey Bay, the Eastern Tropical North Pacific, the Black Sea, Arctic Ocean, and Antarctic coasts (**Figure 7A**) and sediment samples from Elkhorn Slough, and Huntington Beach of California, Bahïa del Tbóari of Mexico, San Francisco Bay, and the tropical West Pacific Continental Margin and a Oak Ridge soil (**Figure 7B**).

## DISCUSSION

### OCCURRENCE OF THAUMARCHAEOL IN HYPERSALINE QINGHAI–TIBETAN LAKES

Thaumarchaeol was observed from the waters and sediments of Gahai Lake2, Xiaochaidan Lake, and Lake Chaka (salinity: 84, 160, and 325 g L^-^^1^, respectively). Their salinities were much higher than those in other lakes where thaumarchaeol has been reported (Blaga et al., 2009; Sinninghe Damsté et al., 2009; Bechtel et al., 2010; Tierney et al., 2010; Pearson et al., 2011; Buckles et al., 2013). Many previous studies indicated that thaumarchaeol is a specific membrane lipid biomarker of Thaumarchaeota (Pearson et al., 2004; Zhang et al., 2006; Pester et al., 2011; Pitcher et al., 2011a; Sinninghe Damsté et al., 2012). So far, thaumarchaeol has not been discovered in other archaea suggesting that thaumarchaeol may be used as a characteristic tracer for Thaumarchaeota in the environment. A possible explanation for the occurrence of thaumarchaeol in such high-salinity lakes is that some AOA in Tibetan lakes may have adapted to higher salinity than that of seawater. This possibility was supported by the presence of archaeal *amoA* gene in these lakes, except for the Lake Chaka (325.0 g L^-^^1^) where the *amoA* gene could not be amplified. Indeed, the hypersaline Lake Chaka is optimal for halophilic Euryarchaeota but not for Crenarchaeota/Thaumarchaeota (Jiang et al., 2006, 2007; Auguet et al., 2009). However, we observed higher PL thaumarchaeol concentration in the Lake Chaka sediment than in other hypersaline lakes. The inconsistency between *amoA* gene result and thaumarchaeota data in Lake Chaka could be ascribed to the following possible reasons: (1) different detection limits between these two methods (qPCR vs. lipid biomarker); (2) preferential degradation of DNA/RNA relative to lipid (Castañeda and Schouten, 2011; Schouten et al., 2013). PL GDGTs may contain a number of GDGTs that have sugar or phosphate groups, and a majority of the PLs (especially glycolipids) in environmental samples might be a result of the selective preservation of fossil lipids (Schouten et al., 2010); (3) the PCR primers used in this study may be limited to amplify all AEA *amoA* genes. For example, the *amoA* genes from genus *Nitrosocaldus* cannot be detected with the primers used in this study (de la Torre et al., 2008); (4) additional sources of thaumarchaeota other than AEA; (5) Transport of thaumarchaeota from surrounding soils by rain runoff and dust particles by wind, where *amoA* gene may have been preferentially degraded. Lake Chaka is a small and shallow lake that is surrounded by soil. When PL GDGTs from surrounding soil was washed into the lake, it can quickly deposit into sediment. GDGTs are difficult to degrade even under oxic conditions (Kim et al., 2009) and thus they can be preserved in sediments for million years (Schouten et al., 2013). However, further investigation is required for the exact reasons for the presence of high GDGT in the Qinghai–Tibetan hypersaline lakes.

### AEA COMMUNITY COMPOSITION IN QINGHAI–TIBETAN LAKES AND THEIR RESPONSE TO ENVIRONMENTAL CHANGES

In recent years, there have been numerous studies to reveal AEA diversity and community composition in freshwater lakes and their relationships with environmental conditions (Herrmann et al., 2009; Pouliot et al., 2009; Llirós et al., 2010; Wu et al., 2010; Auguet et al., 2011, 2012; French et al., 2012a; Auguet and Casamayor, 2013; Hugoni et al., 2013; Vissers et al., 2013). However, systematic AOA studies in saline/hypersaline lakes are still sparse, except for Qinghai Lake and some high-altitude lakes (Jiang et al., 2009b; Hu et al., 2010), likely due to the fact that no AOA cultures have ever been obtained from (hyper)saline environments with salinity higher than that of seawater. Life in hypersaline environments is energetically expensive because microorganisms need to cope with osmotic stress (Oren, 2011). Although ammonia oxidation is an energy-yielding process (Könneke et al., 2005), the known AOA are often limited to those environments with salinities less than that of seawater, because of this energetic consideration. Thus, it is not surprising to retrieve archaeal *amoA* genes from Erhai and Gahai Lake 1 because of their low salinities (<seawater salinity). However, it was unexpected to retrieve archaeal *amoA* genes from hypersaline Gahai Lake2 and Xiaochaidan Lake. Interestingly, cDNA-based archaeal *amoA* gene PCR was successful for the sediments of Xiaochaidan Lake and the resulting *amoA* gene sequences were different from DNA-based sequences. This suggested that AEA might be active in hypersaline lakes with a salinity up to 160 g L^-^^1^. In addition, the majority of the AEA *amoA* gene clone sequences obtained from these lake water samples were affiliated with the “low salinity” cluster, and were widely distributed in different lakes, suggesting that some low-salinity AEA may have adapted to a wide salinity range in Qinghai–Tibetan lakes. In addition, the amino acid sequences from the *amoA* gene clones of this study were highly similar to one other, suggesting that unique environmental conditions (high elevation, strong ultraviolet and dry climate) of the Qinghai–Tibet Plateau may have limited the AEA community diversity in these lakes to a low level.

Many studies have suggested that AOA (Fernàndez-Guerra and Casamayor, 2012) ecological niches are affected by various environmental factors, such as dissolved oxygen (DO), temperature, and salinity (see review by Erguder et al., 2009 and references therein). In this study, salinity and other water chemistry did not have significant correlations with AEA community diversity. However, the difference was observed in the AEA communities between waters and sediments (**Figure 3**), suggesting that the AEA in the sediments were native, and they were not derived from the water column. This observed difference between water and sediment was consistent with previous studies (Francis et al., 2005; Beman and Francis, 2006; Jiang et al., 2009b) and may be ascribed to the fact that water and sediment are different habitats with different environmental conditions. However, with limited data, it is not realistic to identify which factor accounts for the observed difference in the *amoA* gene communities between water and sediments.

Furthermore, the AEA community composition in the studied Qinghai–Tibetan lakes was different from those in other lakes (**Figure 6**) and saline environments (**Figure 7**) worldwide, suggesting that the Qinghai–Tibetan lakes are a unique habitat (e.g., high elevation, strong UV exposure, and dry climate) and thus AEA in these lakes may possess different evolutionary history than their counterparts in other ecosystems.

### COMPARISON BETWEEN THE TWO PRIMER SETS FOR AEA *amo*A GENES

The primer set of CrenamoA23f/CrenamoA616r was originally designed by Tourna et al. (2008) on the basis of the soil fosmid 54d9 (Treusch et al., 2005) and the Sargasso Sea data set (Venter et al., 2004). Subsequently it has been used to successfully amplify AOA *amoA* genes from soil samples (Tourna et al., 2008; Hallin et al., 2009; Stopnivšek, 2010; Yao et al., 2011; Zhang et al., 2012). However, in this study higher AEA *amoA* gene diversity was obtained with the use of the primer set of Arch-amoAF/Arch-amoAR (Francis et al., 2005). Furthermore, non-singleton OTUs derived from the CrenamoA23f/CrenamoA616r primer set were already present within the clone libraries constructed from the Arch-amoAF/Arch-amoAR primer set (**Figure 5**). These lines of evidence suggest that the primer set of Arch-amoAF/Arch-amoAR is more appropriate than CrenamoA23f/CrenamoA616r in characterizing the AEA diversity in the Qinghai–Tibetan lakes.

## Conflict of Interest Statement

The authors declare that the research was conducted in the absence of any commercial or financial relationships that could be construed as a potential conflict of interest.

## Footnotes

2 http://www.mbio.ncsu.edu/bioedit/bioedit.html

3 http://www.ncbi.nlm.nih.gov

4 http://folk.uio.no/ohammer/past/
